# Dynamic Bonds in Biopolymers: Enhancing Performance and Properties

**DOI:** 10.3390/polym17040457

**Published:** 2025-02-09

**Authors:** Trong Danh Nguyen, Jun Seop Lee

**Affiliations:** Department of Materials Science and Engineering, Gachon University, 1342 Seongnam-Daero, Sujeong-gu, Seongnam-si 13120, Gyeonggi-do, Republic of Korea; ntdanh041@gachon.ac.kr

**Keywords:** bio-generated polymers, bio-decomposable polymers, dynamic covalent bonds, hydrogen bonds, drug delivery

## Abstract

As the demand for polymer materials increases, conventional petroleum-based synthetic polymers face several significant challenges, including raw material depletion, environmental issues, and the potential for biotoxicity in biological applications. In response, bio-based polymers derived from natural sources, such as cellulose, alginate, chitosan, and gelatin, have garnered attention due to their advantages of biocompatibility and biodegradability. However, these polymers often suffer from poor physical stability due to the high density of hydrogen bonds and the large structure of pyranose rings. This review explores the potential of incorporating dynamic covalent bonds into biopolymers to overcome these limitations. The chemical structures of biopolymers contain numerous functional groups that can serve as anchoring sites for dynamic bonds, thereby enhancing the mechanical properties and overall stability of the polymer network. The review discusses the performance improvements achievable through dynamic covalent bonds and examines the future potential of this technology to enhance the physical properties of biopolymers and expand their applicability in biological fields.

## 1. Introduction

Biomaterials are produced from resources obtained from natural environments. Within these materials, polysaccharides, lipids, and proteins can form polymer chains through biosynthetic processes [1]. Naturally sourced materials include bio-based polymers such as chitosan, cellulose, alginate, and gelatin, each exhibiting unique properties due to their distinct molecular structures. Because these materials are derived from nature, they are not only easy to harvest through routine agricultural activities but also offer biocompatibility [2]. These features highlight their potential applications in the pharmaceutical and healthcare fields. Additionally, their biodegradability makes them suitable for medical applications such as drug delivery and wound healing [3].

However, a major drawback of these bio-based polymers is the inability to control intermolecular hydrogen bonding caused by strongly polar functional groups in their molecular structures [4]. Excessive hydrogen bonding reduces the flexibility of the polymer structures and induces brittleness, which limits their applicability across various fields. To address these challenges, covalent bonds have been introduced between polymer chains to facilitate the formation of stable networks. The presence of covalent crosslinks enhances the stability of these networks and prolongs the decomposition time of bio-based polymer materials. Furthermore, the improved mechanical properties resulting from these covalent interactions allow the materials to be used in applications such as packaging, wound healing, tissue regeneration, biodegradable coatings, and films [5,6,7].

Recently, research has focused on using dynamic covalent bonds, which can reversibly break and reform in response to external stimuli, to form polymer networks instead of relying on irreversible covalent crosslinks [8]. Dynamic covalent bonds improve the performance of polymer materials by enhancing properties such as toughness, stretchability, adhesiveness, stability, sensitivity to environmental changes, and self-healing.

**Figure 1:**
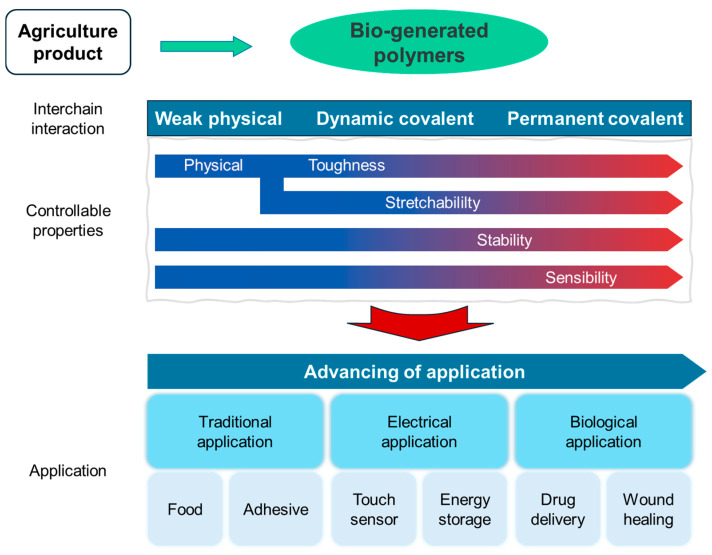
The introduction of dynamic covalent bonds to the network of biological polymers and their advanced applications.

This review discusses the dynamic bonds used to improve the properties of bio-based polymer materials for biological applications (Figure 1). Specifically, it introduces commonly used bonds such as imine and boronate linkages, as well as disulfide bonds, which can undergo reversible structural changes under low energy, and Diels–Alder bonds, which can be modulated by light wavelengths. Furthermore, this review proposes potential biological applications of bio-based polymers incorporating these dynamic bonds, including drug delivery systems and bioadhesive materials.

## 2. Dynamic Covalent Bonding

Similar to hydrogen bonds, certain covalent bonds exhibit dynamic properties. These bonds can undergo cycles of bonding and debonding, but specific conditions must be met for these processes to occur [9,10,11]. Since covalent bonds between identical molecules are more stable than non-covalent interactions, suitable energy, catalysts, hydrogen ion potential (pH), or physical forces are required to activate dynamic bonding [12,13,14,15,16,17]. Table 1 compares the energy levels of dynamic covalent bonds with those of permanent covalent bonds and weak physical interactions. Additionally, the conditions required to trigger the reversible reactions of dynamic covalent bonds are outlined.

In terms of energy, the reverse reactions that break dynamic bonds occur when sufficient energy is supplied to initiate the process. This energy can often be provided in the form of heat, which is easily accessible in daily life, making polymers with dynamic covalent bonds promising candidates for self-healing materials [18]. Furthermore, some molecules, such as anthracene, can interact with ultraviolet (UV) light, receiving energy through efficient UV irradiation. This allows their reversible reactions to be triggered by UV light alone, without the need for additional energy sources like heat [19,20].

For pH-dependent dynamic covalent bonds, the reversible reaction operates similarly to a typical equilibrium-driven process, where changes in pH shift the equilibrium toward bonding or debonding. A similar mechanism applies to catalyst-activated dynamic bonds, where the presence of a catalyst facilitates the reaction [21]. Different types of dynamic covalent bonds exhibit varying bonding strengths, making the selection of an appropriate bond type crucial for designing a desired polymer network. The chemical structure of molecules forming dynamic bonds influences the morphology and properties of the resulting polymers and must be carefully considered [22].

The incorporation of dynamic covalent bonds introduces advantages such as self-healing, shape memory, recyclability, stress relaxation, and reversible polymerization. To date, various dynamic covalent bonds have been proposed to meet the requirements of diverse conditions, thereby enabling a wide range of applications [23,24]. This review focuses on dynamic covalent bonds such as imine, boronate ester, oxime, acyl hydrazone, disulfide, and Diels–Alder bonds. Although other types of dynamic covalent bonds, such as thioester and anthracene bonds, exist, they are not covered in this section due to their limited use in research on bio-based polymers.

**Table 1:** The energy range of the dynamic covalent bonds in comparison to permanent covalent bonds and weak physical interactions.

Type of Interaction	Bonding	Energy Range (kJ mol^−1^)	Reversible Reaction-Activated Condition	Refs.
Permanent covalent bonds	Carbon–Carbon	300–450		[25,26,27]
	Carbon–Nitrogen	300–430		
	Carbon–Oxygen	280–370		
	Carbon–Silicon	220–310		
	Silicon–Oxygen	420–570		
Weak physical interaction	Hydrogen	0.8–167	pH	[28,29]
	Electrostatic	5–200		[30]
	Coordination	100–300		
	Hydrophobic	0.1–20		
	π-π	1–50		[31]
Dynamic covalent bonds	Imine	40–80	pH, water	[32]
	Oxime	67–130	pH, water	
	Acylhydrazone	45–85	pH, water	
	Boronate ester	27.2–93.3	Water	[33]
	Disulfide	140–290	Temperature	[34]
	Dial-alder	130–350	Temperature	[35,36]

### 2.1. Imine Bonds (Schiff Base Reaction)

**Figure 2:**
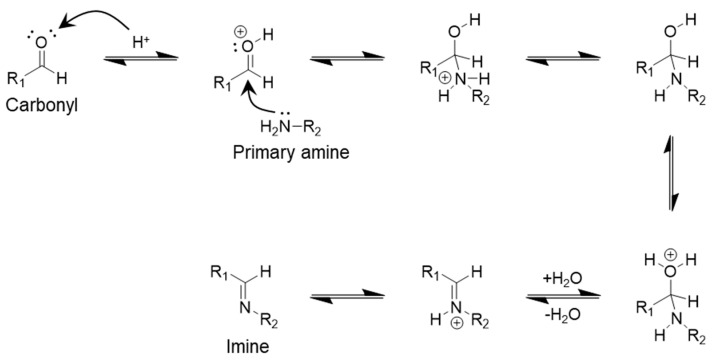
Reaction showing the formation of imine dynamic covalent bond.

Imine dynamic covalent bonds are among the most commonly introduced dynamic bonds in biopolymer networks. The conditions required for their formation are straightforward and primarily influenced by the pH of the polymer [37]. Imine bond formation is essentially a condensation reaction between an amine group (–NH_2_) and a carbonyl group (C=O, which may be a ketone or an aldehyde), resulting in the release of water molecules (Figure 2) [38].

An acidic environment is particularly favorable for the formation of imine bonds, leading to water being trapped within the network structure [39]. To reverse this reaction, an appropriate pH level must be provided. Imine bonds are stable under neutral conditions but can dissociate into amine and carbonyl groups in mildly acidic or basic environments [40]. Additionally, the amount of water present in the network plays a critical role in determining the direction of the reaction [41]. Therefore, both pH and water content must be carefully considered when designing polymer materials.

The functional groups necessary for imine bond formation can originate from the polymer itself. Specifically, the use of biopolymers in network materials is advantageous due to their abundance of amine functional groups. On the other hand, the introduction of carbonyl groups can be achieved by generating aldehydes through the oxidation of polysaccharides. This process leverages the inherent structure of polysaccharides, typically employing sodium periodate (NaIO_4_) to oxidize the monomer structure, resulting in the opening of the ring at carbon positions 2 and 3 [42,43]. While aldehyde-containing chemicals can also be used, the oxidation of polysaccharides is a more suitable method for creating bio-based and biocompatible polymers.

However, a notable limitation of imine bonds is their reversible bonding and debonding reactions, which are highly sensitive to external environmental conditions, making them relatively unstable.

### 2.2. Boronate Ester Bonds

**Figure 3:**
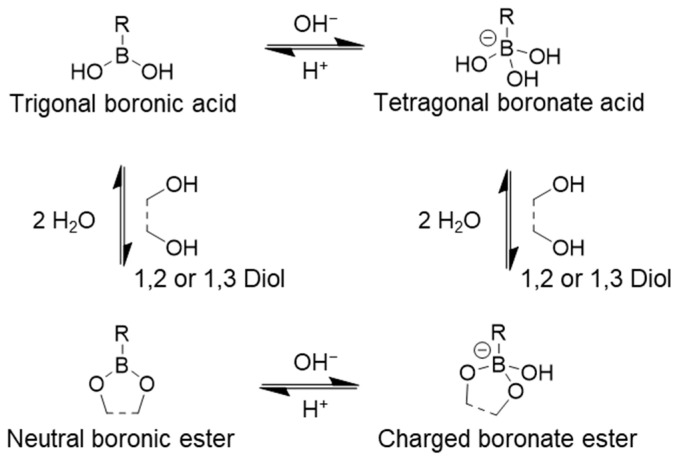
The formation reaction of boronate ester dynamic covalent bond.

The chemical structure of bio-derived polymers contains a high number of hydroxyl functional groups, which serve as the foundation for the formation of boronate ester dynamic bonds. Similar to imine functional groups, boronate ester formation is a condensation reaction that is highly influenced by environmental conditions, including pH and the concentration of water in the network [44,45,46]. Trigonal boronic acid contains hydroxyl groups that can react with adjacent hydroxyl groups on the bio-derived polymer material (Figure 3) [47].

The difference in the number of bonds formed by a single boronic acid can affect the mobility of the polymer chains within the network. However, the strength of boronate bonds is still considered weaker due to the lower negative charge density. Because the conditions for triggering the reversible reaction are similar, both dynamic covalent bonds, imine and boronate, have been used together to facilitate control over the mechanical properties. The key difference is that the bond is stable only at neutral pH, while boronate dissociation occurs under both acidic and basic conditions [48].

The pH sensitivity of the boronate bond is stronger than that of the imine bond, making it more suitable for drug delivery, as pH varies across different areas of the body. Boronic acid, the reactant, must be sourced from another chemical. However, boronic acid-containing compounds are biocompatible, ensuring the potential for bioapplications of the synthesized material [49].

### 2.3. Oxime Bonds

**Figure 4:**
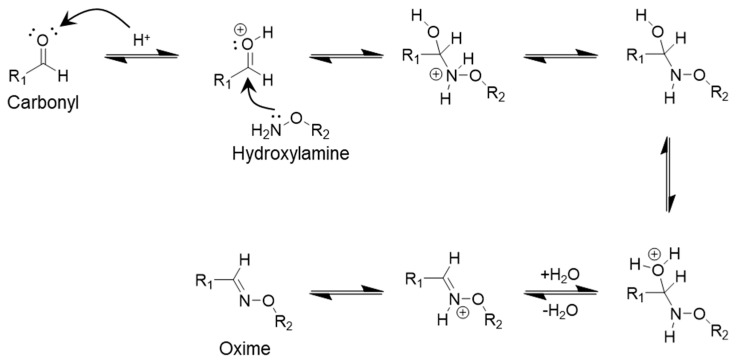
Reaction showing the formation of oxime dynamic covalent bond.

Oxime dynamic covalent bonds are the driving force behind imine bonds. In this case, the link is formed by the condensation reaction between aldehyde and hydroxyl-amine (−O−NH_2_) functional groups (Figure 4) [50,51]. Acidic and basic conditions also trigger reversible reactions of oxime covalent bonds. Although weakly acidic pH (4–6) favors the formation process, stronger acidic or basic conditions can efficiently degrade the dynamic bonds [52]. Under strong acidic conditions, oxime bonds become protons and break easily. On the other hand, strongly basic deprotonated hydroxylamine promoted the hydrolysis reaction [53].

### 2.4. Acylhydrazone Bonds

**Figure 5:**
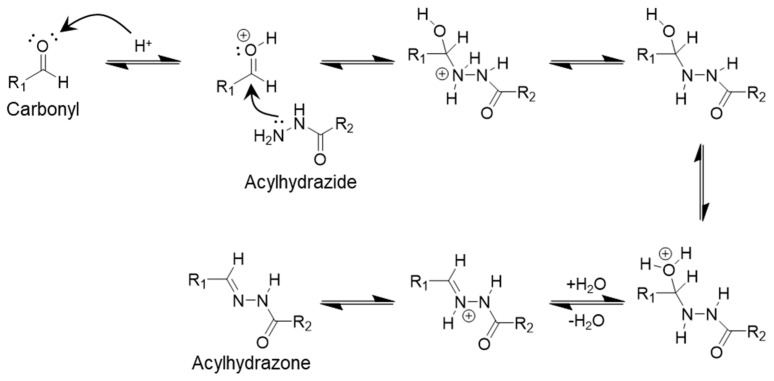
Reaction showing the formation of acylhydrazone dynamic covalent bond.

Acyl hydrazone is a dynamic covalent bond formed through a condensation reaction between acyl hydrazide and an aldehyde or ketone (Figure 5). This reaction was first proposed by the Sander group [54]. While similar to imine dynamic bonding, acyl hydrazone dynamic bonds have been shown to be more stable with respect to changes in material pH. The formation of these dynamic bonds is favored at a pH between 7 and 9, whereas the reverse reaction typically requires a strongly acidic pH (below 4) [55,56]. However, their bioapplications are limited. Several factors promote the dissociation of the acyl hydrazone bond, including water concentration, temperature, and the presence of a catalyst.

### 2.5. Disulfide Bonds

**Figure 6:**
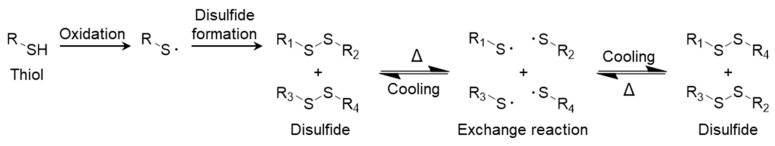
Reaction showing the formation of disulfide dynamic covalent bond.

Dynamic covalent disulfide bonds exist between two sulfur atoms in the chemical structure of a material [57,58]. This linkage is formed through the oxidation of thiol groups, where two hydrogen atoms are removed, creating free radical sulfides that can then pair up and bond (Figure 6) [59]. Unlike imine and boronate bonds, disulfide bonds are stable with respect to changes in pH and water content within the material. The reversible reaction of a disulfide bond does not involve complete bond cleavage. Instead, disulfides become unstable upon the input of energy (e.g., heat or UV light). This triggers a disulfide exchange reaction, where a pair of sulfur atoms partially dissociates into free radical sulfur atoms, acquiring high mobility [60,61]. These free radicals can then attack other disulfide bonds, swapping positions. Upon returning to room temperature, the disulfide bonds stabilize and reform randomly, creating new dynamic disulfide bonds. This reaction provides a simple mechanism for self-healing in polymer materials containing disulfide bonds. However, this process is time consuming and requires an external source of heat or UV light.

Methods have been developed to enable disulfide exchange reactions at lower temperatures. These methods involve the use of reducing agents such as dithiothreitol, 2-mercaptoethanol, and tris(2-carboxyethyl)phosphine [62,63]. These agents donate electrons, cleaving the disulfide bond and generating two thiolate anions (-S^−^). These anions can then reform a disulfide bond with another thiolate or attack a different disulfide bond. Although this process is simpler, the presence of a catalyst raises concerns about application limitations, as it may negatively impact the material’s properties and stability.

### 2.6. Diels–Alder Bonds

**Figure 7:**
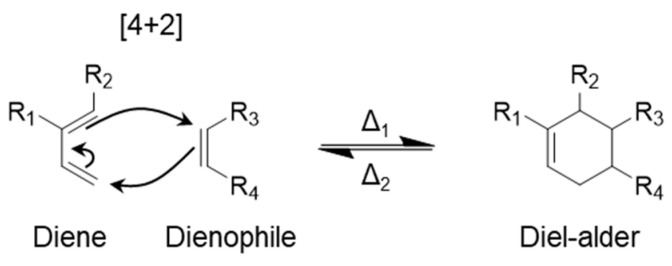
Reaction showing the formation of Diels–Alder dynamic covalent bond.

Diels–Alder dynamic covalent bonds are formed through a [4+2] cycloaddition reaction, resulting in a six-membered ring structure (Figure 7) [64]. This reaction occurs between a dienophile and a diene (a molecule with two conjugated double bonds) and is driven by electronic effects [65]. One reactant acts as an electron-withdrawing group, while the other acts as an electron-donating group. The specific role of each reactant is determined by its functional groups. The diverse range of functional groups leads to varying electronegativities, which in turn have opposing effects on the organic compounds. The presence of electron-donating groups, such as hydroxyl (-OH), ketone (-OCH_3_), and amine (-NH_2_), can make a reactant an electron-donating substituent. Conversely, the presence of electron-withdrawing groups, such as aldehyde (-C=O), cyano (-C≡N), and nitro (-NO_2_), will cause the reactant to act as an electron-withdrawing substituent [66,67]. Extensive research has explored potential reactant pairs for the Diels–Alder reaction, and many of the well-established pairs can be found in the book by Taticchi [68,69].

Temperature is a crucial factor in the reversibility of Diels–Alder bonds. Since the reaction is exothermic, the product is more stable than the reactants [70]. The reverse reaction requires a higher temperature than the forward reaction. Consequently, the dissociated functional groups (dienes and dienophiles) can reform dynamic bonds during the cooling process. This makes it challenging to achieve a network with fully dissociated functional groups. An effective method for facilitating self-healing in Diels–Alder-containing networks involves maintaining the material at a temperature that sustains the dissociated state. Subsequently, the material is held at a temperature conducive to Diels–Alder formation for a specific period. However, this approach has the disadvantage of requiring significant energy input to maintain the elevated temperature for an extended duration.

## 3. Multiple Dynamic Bonds Containing Biopolymers

**Figure 8:**
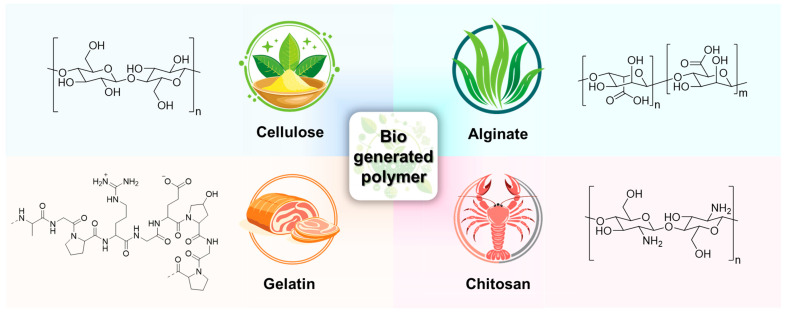
Bio-generated polymers that are collectible from natural sources, including cellulose, alginate, chitosan, and gelatin.

Bio-generated polymers are attracting significant interest in advanced applications such as drug delivery, tissue engineering, wound healing, and healthcare due to their biodegradability, environmental friendliness, and sustainability [71,72]. Unlike conventional polymers derived from crude oil, bio-derived polymers can be obtained from a variety of sources, including tree trunks, insect bodies, wool, and silk (Figure 8). However, these polymers have limitations due to the high production costs associated with low production efficiency and the need for pretreatment before they can be used in actual production processes [73,74,75]. In addition, most bio-generated polymers form hydrogen bonds derived from a large number of polar functional groups, and no clear method for improving their properties has been presented to overcome this. Therefore, processes to overcome these limitations and reduce the processing cost of materials have been continuously attempted. Recently, research has been reported on replacing hydrogen bonds with dynamic covalent bonds to form new polymer networks as a solution to this problem [76,77,78]. This helps bio-generated polymers have high stretchability and self-healing ability due to the combination of hydrogen bonds and dynamic covalent bonds.

### 3.1. Cellulose

Cellulose, a common bio-derived polymer obtained from biomass, can be continuously produced from biowaste generated after each harvest season [79,80]. Cellulose-based materials offer the advantage of not only improving the income of local residents by providing a method of utilizing biowaste but also reducing environmental pollution [81,82]. Through various modifications, this material forms various types of cellulose structures. The most commonly used materials are carboxymethyl cellulose and carboxyethyl cellulose, which, due to their water solubility, can be further modified for advanced biological applications. However, due to its basic structure having many hydroxyl groups, cellulose has many hydrogen bonds in its chemical structure, thus exhibiting high crystallinity [83,84]. The crystallized structure not only makes the polymer brittle but also has limitations in stability.

**Figure 9:**
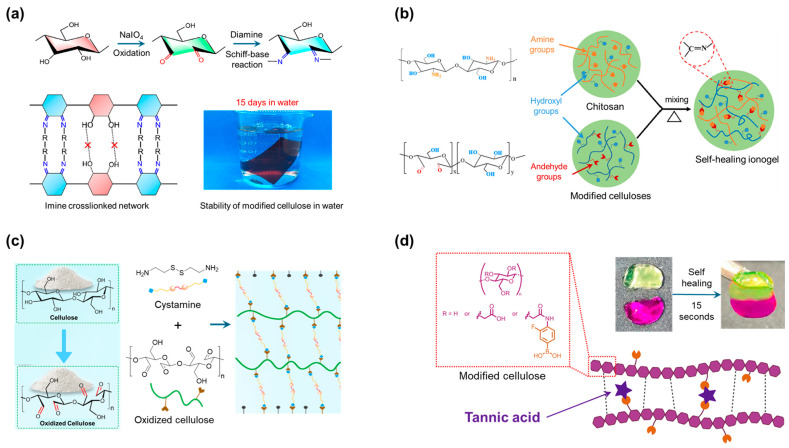
(**a**) Cellulose-based imine dynamic covalent crosslinked polymer through the formation of the aldehyde functional groups [85]. Copyright 2023, American Chemical Society. (**b**) Imine dynamic covalent crosslink between chitosan and cellulose materials [86]. Copyright 2020, Springer Nature. (**c**) The wood adhesive materials are built from celluloses, which are crosslinked with the imine and disulfide dynamic covalent bonds [87]. Copyright 2024, Elsevier. (**d**) High-efficiency self-healing cellulose polymer gel materials built from the boronate crosslinked between cellulose and tannic acid [88]. Copyright 2024, American Chemical Society.

Recently, research has been conducted to overcome these shortcomings. First, Su et al. reconstructed the network through the oxidation of cellulose followed by a Schiff-base reaction (Figure 9a) [85]. The oxidation product is an opened pyranose ring at the positions of carbons 2 and 3, creating two aldehyde functional groups for the formation of aldehyde cellulose. These reactants form dynamic imine covalent bonds through a Schiff-base reaction with amino-terminated polymer chains (priamine). The authors suggested that the introduction of large-sized priamines intercepted and prevented hydrogen bonding between the cellulose chains. It should be noted that the ratio between the cellulose segment and the priamine plays a decisive role in the properties of the generated network, as the extra (unreacted) carbonyl was proposed to be able to produce hydrogen interactions and strengthen the structure. Wang et al. used the same approach to generate aldehyde cellulose as a base for forming dynamic imine covalent bonds (Figure 9b) [86]. Dynamic bonding was created between the aldehyde and amino groups of chitosan, which acted as crosslinkers, leading to the formation of a copolymer material that exhibited excellent thermomechanical stability.

Yin et al. used an oxidation process to synthesize aldehyde cellulose (Figure 9c) [87]. In this study, imine bonds were created with cystamine. Thus, covalent disulfide bonds were introduced into the network. In the disulfide exchange reaction, the polymer network functions as an adhesive material. The author also performed an oxidation process to generate aldehyde groups for imine dynamic bonds while preparing for the application. Instead of cellulose, Ollier et al. proposed a material based on carboxymethylcellulose, which resulted from the introduction of carboxymethyl groups onto cellulose (Figure 9d) [88]. Subsequently, the material was modified with 4-amino-3-fluorophenylboronic acid, which was able to generate boronate ester covalent bonds with tannic acid. Tannic acid also generated hydrogen bonds, which maintained the softness and self-healing ability of the material.

### 3.2. Alginate

Alginate is a polysaccharide primarily derived from algae such as Macrocystis, Laminaria, and Ascophyllum [89]. One of the most significant advantages of alginate is its biodegradability, which makes it a promising material for biological applications [90,91]. The chemical structure of alginate is built from two monomers, mannuronic acid and guluronic acid, as block copolymers. Depending on the copolymer source, the arrangement of the two monomers can change and influence the properties of the material [92,93]. Although the source of alginate is rich owing to the easy farming method for algae, its poor mechanical properties limit its use in a variety of applications. Because of the strong hydrogen bondings, alginate is brittle, even when it is in the gel state. Thus, considerable research has been conducted to enhance the connections between the polymer chains through the formation of dynamic covalent bonds.

**Figure 10:**
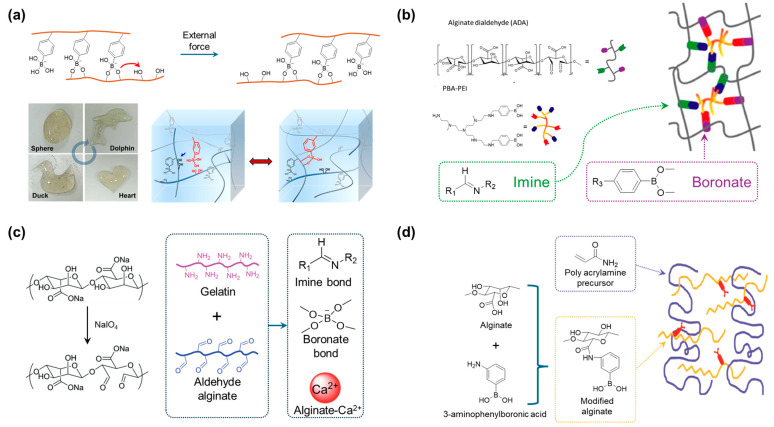
(**a**) The alginate-based multiple dynamic functions polymeric hydrogel crosslinked by boronate covalent bond [94]. Copyright 2018, American Chemical Society. (**b**) The dual dynamic covalent crosslinked network of boronate and imine for the application of antibacterial materials [95]. Copyright 2022, American Chemical Society. (**c**) A highly hydrophilic Ca^2+^-contained three-dimensional network with a large amount of water [96]. Copyright 2020, Royal Society of Chemistry. (**d**) Smart hydrogel with multiple functionalities with three categories of stimulus [97]. Copyright 2018, John Wiley and Sons.

Hong et al. introduced a phenylboronic functional group by modifying alginate via the reaction between the amine of 3-Aminophenyl boronic acid and the hydroxyl group of alginate (Figure 10a) [94]. The reaction occurred in the presence of an N-hydroxysuccinimide/1-(3-(dimethylamino)-propyl)-3-ethylcarbodiimide hydrochloride (EDC/NHS) catalyst, which is well known for its high yield. The functional groups formed dynamic bonds with the remaining hydroxyl groups of the alginate. These bonds were proposed to dissociate and reform the new connection with another hydroxyl group, and the polymer was affected by an external force stimulus. Thereafter, the modulus of the material could be controlled by adding glucose and triggering a dynamic reaction. Shen et al. introduced an alginate-based material containing multiple dynamic covalent bonds (Figure 10b) [95]. First, a portion of the alginate monomer was oxidized with NaIO_4_ (approximately 10% oxidation degree) to generate aldehyde functional groups, which are the basis for the formation of imine dynamic bonding. On the other hand, a multifunctional crosslinker includes an amine (for imines) and boronic acid (for boronate esters). The structure of the gel material can be tuned by varying the ratio of the functional groups of the crosslinkers. The properties of the material could be further controlled by the introduction of glucose, similar to the mentioned previous research of Zhang et al., who synthesized the oxidized alginate, which forms an imine bond with amine-rich gelatin (Figure 10c) [96]. In this study, boronate ester bonds were formed by the introduction of borax, and Ca^2+^ formed an egg-box structure. The focus of this study was to investigate the influence of Ca^2+^ on the mechanical properties of the materials. As the ion concentration increased, the stress at break, strain at break, adhesion, and self-healing ability of the material improved.

Zhang et al. introduced an alginate base gel material reinforced with polyacrylamide (Figure 10d) [97]. 3-aminophenylboronic acid was first grafted onto alginate in the presence of a catalyst. The modified alginate was mixed in water with the acrylamide monomer, which later underwent polymerization to ensure excellent dispersity of the two polymers. The immersion of the material in a solution of CaCl_2_ and NaOH was carried out to form the bond of the boronate ester and introduce Ca^2+^, which had an impact on the properties of the polymer. The material can respond to multiple triggers such as moisture, multivalent cations, and pH, making it suitable for applications in soft robotics.

### 3.3. Chitosan

Chitosan is a biopolymer found in natural sources such as the exoskeleton of crustaceans, crabs, and shrimp [98,99]. Although chitosan is biodegradable, its degradation can take a few months to several years, depending on the environmental conditions [100]. Chitosan has been used in medical, pharmaceutical, and agricultural applications due to its excellent biocompatibility, antimicrobial activity, cholesterol reduction, fat-binding properties, and good tensile strength [101,102]. Currently, active research is being conducted on controlling the physical properties of chitosan by introducing various dynamic covalent bonds to further expand its applications.

**Figure 11:**
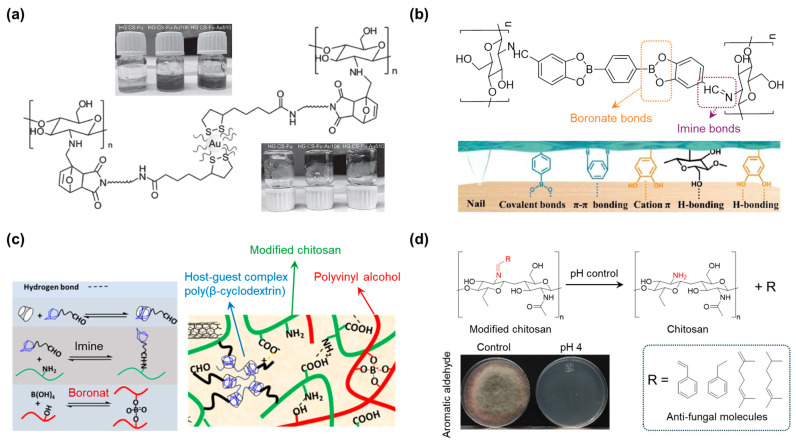
(**a**) Research on nanoparticle crosslinkers and click chemistry for creating nanocomposite hydrogels [103]. Copyright 2016, John Wiley and Sons. (**b**) Biomass wood glue is made from chitosan oligosaccharides with multiple interface interactions that are recyclable, robust, and environmentally friendly [104]. Copyright 2024, Elsevier. (**c**) Electrical conductive/tissue adhesive hydrogel for the applications of human and organ monitoring [105]. Copyright 2021, Elsevier. (**d**) pH-responsive antifungal films synthesized using reversible covalent chemistry of imines and different naturally occurring aldehydes [106]. Copyright 2022, Elsevier.

Garcia-Astrain et al. formed a uniform composite of chitosan and maleimide-coated gold nanoparticles using Diels–Alder dynamic bonding (Figure 11a) [103]. Chitosan was modified to carry furan functional groups by reacting its amine groups with acetic acid, followed by reducing the Schiff base formed during the reaction. This modification allowed the gold nanoparticles to form strong covalent bonds with chitosan, preventing the negative impact of additional fillers, as demonstrated through rheological investigations.

To introduce chitosan for wood adhesive applications, Yu et al. modified chitosan using an imine dynamic bond reaction between the amine groups of chitosan and 3,4-dihydroxybenzaldehyde (DBA), which contains an aldehyde and two hydroxyl functional groups (Figure 11b) [104]. These hydroxyl groups formed a boronate ester dynamic bond with 1,4-phenylenediboronic acid (PBA). The combination of DBA and PBA generated multiple interactions with the wood interface, including boronate ester covalent bonds, π–π interactions, cation–π interactions, and hydrogen bonding. This material exhibited excellent adhesive properties and maintained adhesion for more than 30 days.

Ren et al. fabricated a complex of supramolecular crosslinkers that provided multiple crosslinking interactions (Figure 11c) [105]. The crosslinker was synthesized based on the host–guest complexation of poly(β-cyclodextrin) (poly(β-CD)) with adamantane, which was able to form imine bonds with chitosan and boronate ester bonds with polyvinyl alcohol. A large number of hydrogen bonds were also present within the network.

Mozos et al. used dynamic covalent imine bonds to modify chitosan with various antifungal materials (Figure 11d) [106]. Specifically, they induced an increase in antifungal activity by controlling the pH of the imine bond between the antifungal agent and the chitosan chain, which in turn controlled the release of the antifungal agent. The direct binding of the aldehyde-containing materials to chitosan also ensured the mechanical properties of the proposed material.

### 3.4. Gelatin

Gelatin is synthesized through a partially hydrolyzed process of collagen, a protein derived from animal connective tissues, including bones, cartilage, and skin [107,108,109]. Gelatin dissolves easily in water and forms a gel structure at room temperature [110]. The gel made from gelatin has stretchability that is lacking in plant-based biopolymer gels. However, gelatin-based gel materials do not meet the mechanical requirements for more advanced applications such as tactile sensors or adhesives [111]. Therefore, many attempts have been made to improve their poor mechanical properties by introducing crosslinkers, and with dynamic covalent bonds, materials can recover their enhanced properties. Their chemical structures have many branches terminated by amines, facilitating various modifications, as they can be the basis for many crosslinking reactions.

**Figure 12:**
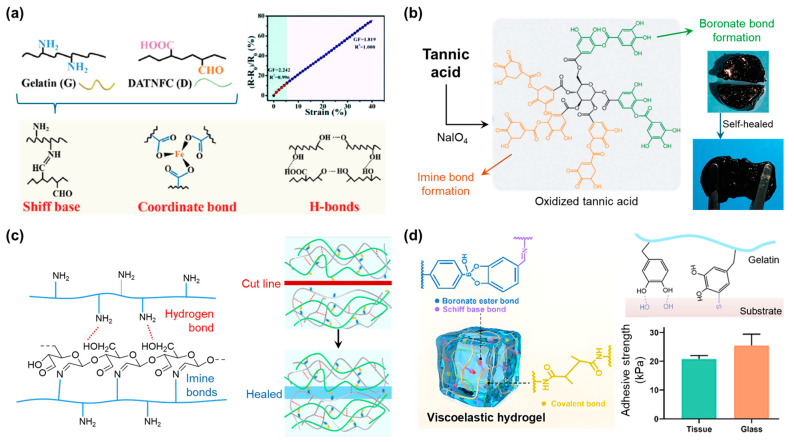
(**a**) Multiple crosslinkers conducting gelatin-based networks for the application of resistive tactile sensors [112]. Copyright 2022, Royal Society of Chemistry. (**b**) A dynamically crosslinked multifunctional hydrogel adhesive that exhibits inherent photothermal properties, injectability, self-healing characteristics, antioxidant activity, and antibacterial capabilities [113]. Copyright 2023, John Wiley and Sons. (**c**) Synthesizing of injectable, self-healable, and biocompatible hydrogel system based on gelatin material [114]. Copyright 2022, Elsevier. (**d**) Strategy for fabricating viscoelastic hydrogels with controllable dynamic mechanical properties [115]. Copyright 2023, Elsevier.

Fu et al. proposed a gelatin-based gel material for applications in tactile sensors (Figure 12a) [112]. The authors introduced multiple types of interactions to overcome the poor mechanical properties. They created dynamic imine covalent bonds between the amine functional groups of gelatin and the aldehyde functional groups of oxidized nanofibrillated cellulose. When Fe^3+^ is present, three types of interchain interactions exist in the network: imine covalent dynamic bonds, coordinate bonds, and hydrogen interactions. The mechanical properties were significantly improved, with the tensile stress being 10 times higher and the compressive stress being approximately 38 times higher. Fe^3+^ also provides electrical conductivity to the gel, which allows the material to be applied as the sensing layer of a resistive tactile sensor, where it exhibits excellent working performance.

Kang et al. introduced oxidized tannic acid into the gelatin chains to form a network structure (Figure 12b) [113]. Tannic acid, oxidized by NaIO_4_, undergoes thermal conversion, which gives it antibacterial ability. On the other hand, the modification of tannic acid converts a large amount of the hydroxyl functional groups in the molecular structure to aldehyde, which forms an imine bond with the amine of gelatin. In addition, borax ions are added to the network to form boronate bonds. As a result, gelatin not only has higher stress and strain at break during the tensile test but also has excellent tissue adhesion and self-healing abilities.

Cui et al. used oxidized cellulose as a source of aldehyde functional groups to form imine dynamic covalent bonds with gelatin (Figure 12c) [114]. Specifically, they controlled the mechanical properties of the resulting material by adjusting the ratio of aldehyde functional groups to amine functional groups. Furthermore, Liu et al. attempted to optimize the mechanical properties of gelatin hydrogel materials (Figure 12d) [115]. The gelatin chains were bound together by covalent and dynamic covalent crosslinkers, and by varying their ratios, they controlled the properties of the material through rheological analysis.

## 4. Dynamic Covalent Bonds in the Biological Application

### 4.1. Drug Delivery Systems

Drug delivery systems (DDSs) are designed to transport therapeutic molecules to specific sites within the bodies of animals or humans. Despite their origins dating back to the 1970s, the primary focus in this field is on achieving controlled release rates [116]. The incorporation of dynamic covalent bonds, which can dissociate under appropriate conditions, endows the material with the capability to release drugs at a precisely controlled rate. Polymers can be interconnected via these dynamic covalent bonds, allowing drugs to be directly loaded into the gel matrix. These dynamic bonds act as crosslinkers within the polymer networks, enhancing the controllability of drug release processes [26]. Upon meeting the conditions for dissociation, the network becomes more unstable, thereby facilitating drug release.

**Figure 13:**
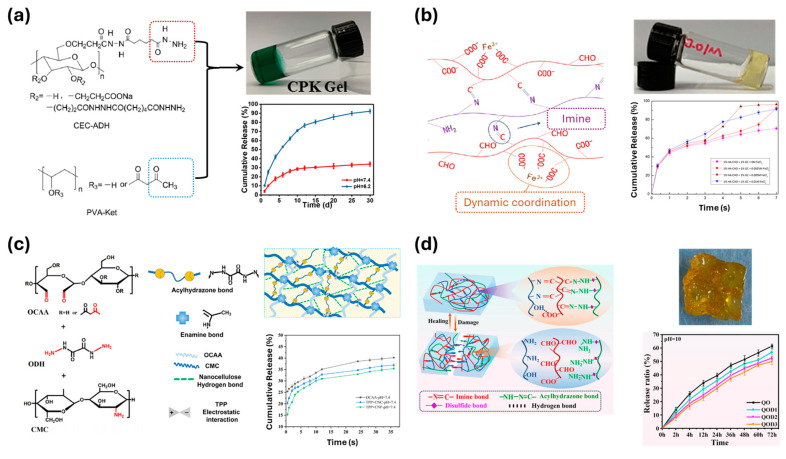
(**a**) Drug delivery system gel materials constructed from modified carboxyethyl cellulose and polyethylene glycol [117]. Copyright 2021, Elsevier. (**b**) Imine-based build from the oxidized hyaluronic acid and glycol chitosan [118]. Copyright 2021, American Chemical Society. (**c**) Dual dynamic covalent crosslink in the network of acetoacetate cellulose and carboxymethyl chitosan [119]. Copyright 2022, Elsevier. (**d**) Multi dynamic crosslink, imine, acylhydrazone, disulfide, and hydrogen bonds, in the drug delivery system [120]. Copyright 2024, Elsevier.

Jiang et al. synthesized ketoester-type acylhydrazone, a variant of acylhydrazone dynamic covalent bonds, between carboxyethyl cellulose and polyvinyl alcohol chains (Figure 13a) [117]. The reversible formation of acylhydrazone bonds can be regulated by pH changes. In an acidic environment, the dynamic crosslinks dissociate, facilitating the release of doxorubicin (DOX). By exposing the DOX-containing sample to different pH conditions, they observed variations in the release rate. While 100% of DOX was released within 30 days in a pH 6.2 buffer, only 34% was released in a pH 7.4 buffer. On the other hand, Gou et al. formed imine bonds between oxidized hyaluronic acid and glycol chitosan with FeCl_3_ incorporation (Figure 13b) [118]. Two factors control the degradation of the hydrogel: acid promotion and the presence of Fe^3+^. Compared to the pristine sample, the sample containing Fe^3+^ exhibited a higher release rate. These results further demonstrate the enhanced ability to control the release rate of dynamic covalent bond-based polymer materials.

Wang et al. adopted an alternative approach by utilizing two different dynamic covalent bonds, imine and acylhydrazone, as crosslinkers between oxidized acetoacetate cellulose and carboxymethyl chitosan (Figure 13c) [119]. Both types of dynamic bonds are sensitive to pH conditions, making them a suitable combination. Additionally, the dual network structure has the potential to enhance mechanical strength, which was further improved by the addition of nanofillers (cellulose nanofibrils and cellulose nanocrystals). However, there remains a lack of control over the ratio of the crosslink types.

Zhang et al. proposed a network where polymer chains (quaternized carboxymethyl chitosan, oxidized hyaluronic acid, and 3,3′-dithiobis-(propionohydrazide) (DTP)) were crosslinked by multiple types of dynamic covalent bonds (Figure 13d) [120]. The polymer network includes four types of crosslinkers: imine, acylhydrazone, disulfide, and hydrogen bonds. By varying the amount of DTP, the ratio of dynamic bonds can be controlled, thereby influencing properties such as the swelling ratio and release rate of the materials. Additionally, the interaction between the amount of DTP and pH conditions was investigated, offering further control over the drug release rate.

### 4.2. Bio-Adhesive, Wound Healinng

In the field of advanced biological applications, biocompatible polymers can be utilized to cover open wounds and support wound treatment [121]. These applications are identified as wound healings or wound healing materials. The primary role of these materials is to act as a barrier that adheres to the skin, with their foremost function being to prevent bacterial invasion [122]. For a material to be suitable for wound healing applications, it must meet requirements for biocompatibility and biodegradability, as well as possess good physical and adhesive properties to withstand the daily activities of patients [123]. Therefore, biopolymer networks with introduced dynamic covalent bonds are highly suitable for this application. These materials not only exhibit improved physical properties but also possess self-healing capabilities. Additionally, the material can be loaded with medications that promote the healing process, necessitating controlled release rates.

**Figure 14:**
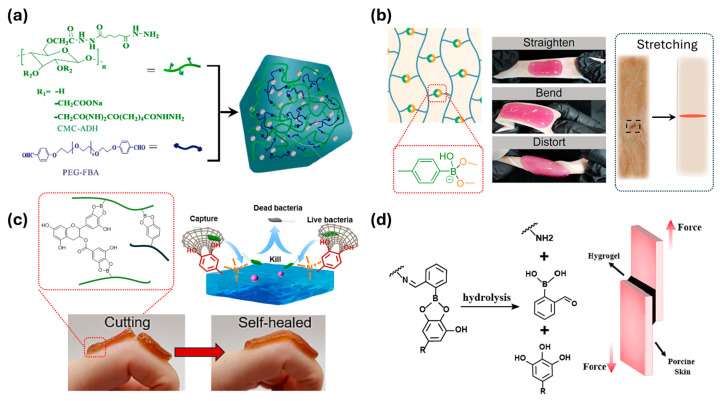
(**a**) Carboxymethyl cellulose-based network containing acylhydrazone dynamic covalent bonds [124]. Copyright 2023, Elsevier. (**b**) Injectable polymer network with boronate ester for the application of wound healing [125]. Copyright 2022, Elsevier. (**c**) Chitosan polymers connect with epigallocatechin-3-gallate wound healing agent through boronate ester bonds [126]. Copyright 2022, Elsevier. (**d**) Adhesive chitosan-based material with the boronate ester dynamic crosslink [127]. Copyright 2024, Elsevier.

Li et al. utilized acylhydrazone dynamic covalent bonds to form a copolymer network between modified carboxymethyl cellulose and 4-formylbenzoic acid-terminated poly(ethylene glycol) (PEG-FBA) (Figure 14a) [124]. The properties of these materials can be effectively controlled by varying the PEG-FBA, which contains aldehyde functional groups. The adhesive strength of the proposed material is recorded to be up to 24 kPa. Additionally, with the well-controlled release of ciprofloxacin, a widely used antibacterial agent, the gel material appears to be a promising candidate for treating irregular wounds and hemorrhages.

In the application of wound healing, injectables are also an important property that can expand the field of wound healing applications to the internal body of the patient. For this purpose, the gelation of the gel material needs to be controlled, which is suitable for the reversible reaction of dynamic covalent bonds. Li et al. constructed the network from hyaluronic acid, which was modified with 3-aminophenylboronic acid (Figure 14b) [125]. This modification provided the polymer with boronic acid functional groups, which are the source of the boronate ester dynamic crosslinks and excellent adhesive properties. The gelation of the material occurred 30 s after the addition of NaOH. The results showed that the proposed gel can stop bleeding more effectively compared to gauze and fibrin glue.

On the other hand, Zhong et al. utilized boronate ester dynamics as crosslinks for chitosan polymer chains (Figure 14c) [126]. The chitosan underwent two different modifications to generate part A with boronic acid and part B with catechol functional groups, which are the reactants for the formation of boronate esters. Additionally, both parts of chitosan were modified with (3-chloro-2-hydroxypropyl) trimethylammonium chloride, which influences the gelation of the material. Consequently, no further pH control is required for the gelation process. Furthermore, boronate ester bonds were also formed with epigallocatechin-3-gallate (EGCG), a well-known healing agent with antimicrobial, antioxidant, and anti-inflammatory properties. Since EGCG contains multiple hydroxyl groups, it is a suitable candidate for forming boronate ester bonds with the main network, thereby enhancing the stability of the material.

Su et al. introduced a multiple dynamic network generated from carboxymethyl chitosan (Figure 14d) [127]. To form the dynamic bonds, 2-formylphenylboronic acid and EGCG were used. The similar formation conditions of imine and boronate ester bonds allow the gel network to form in one pot within 15 s. Since EGCG acts as the crosslinker, varying its concentration leads to changes in the physical properties of the network, including adhesiveness, rheological properties, the drug release rate, and antibacterial ability.

### 4.3. Bioinks for Three-Dimensional Printing

Bioprinting is a technique that leverages advances in both 3D printing and biological materials [128]. This technique has significantly contributed to the fields of tissue engineering, regenerative medicine, and pharmaceutical research. While bio-generated bioinks exhibit good biocompatibility, they often lack the mechanical strength required for certain applications. The introduction of dynamic covalent bonds imparts the necessary mechanical properties to overcome these inherent limitations. Additionally, the water content-dependent reversible reactions of dynamic bonds, such as imine and boronate ester, enable the material to form a stable network post-printing. Consequently, the printed objects demonstrate excellent potential for cell culturing, which holds promise for further development [129].

**Figure 15:**
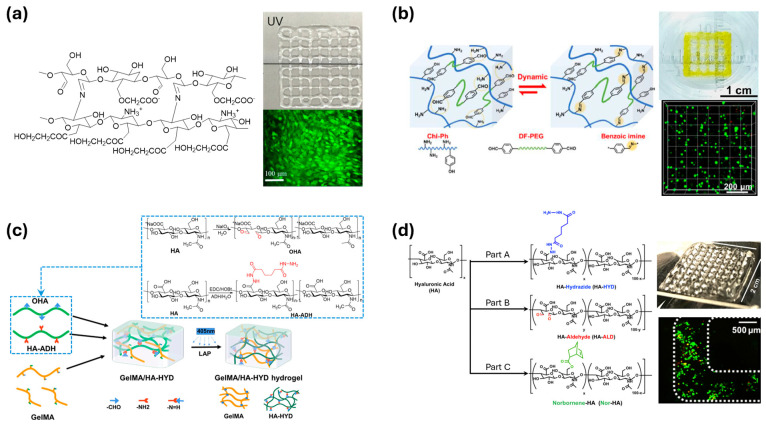
(**a**) Imine dynamic crosslink in the network of carboxymethyl cellulose and glycol chitosan [130]. Copyright 2020, Elsevier. (**b**) Printable chitosan network with the crosslink of imine and a secondary photo-crosslink [131]. Copyright 2021, Elsevier. (**c**) A gel network with imine dynamic covalent bonds and secondary photo-crosslink built from gelatin and hyaluronic acid [132]. Copyright 2022, American Chemical Society. (**d**) Three-dimension printing hyaluronic acid network with acylhydrazone dynamic bonds and secondary photo-crosslink [133]. Copyright 2018, John Wiley and Sons.

Janarthanan et al. proposed a 3D-printable network based on reversible dynamic imine bonds (Figure 15a) [130]. The network was formed from the aldehyde of oxidized carboxymethyl cellulose and the amine of glycol chitosan. Typically, the instability of imine bonds towards pH changes could limit the applications of the printed material. However, the combination of carboxymethyl cellulose and chitosan helps stabilize these pH changes.

Liu et al. introduced printable polymer materials derived from chitosan and polyethylene glycol (Figure 15b) [131]. The PEG was modified with 4-formylbenzoic acid to facilitate the formation of imine bonds. Furthermore, by modifying chitosan with phloretic acid, the irradiation of a blue diode (440–460 nm) can activate a photo-crosslink between the chitosan chains. These act as secondary crosslinks that stabilize the material post-printing.

Wang et al. developed a bioprinting ink from hyaluronic acid capable of generating imine bonds (Figure 15c) [132]. The authors employed a strategy of modifying the polymer into two parts: part A with adipic acid dihydrazide and part B with oxidized polymer. These modified polymers can form dynamic acylhydrazone bonds. Additionally, gelatin methacrylate, which has photo-crosslinking capabilities, was introduced into the network. As the polymers exit the nozzle, they are exposed to UV light, forming a stable structure. Wang et al. conducted a similar modification of hyaluronic acid, but instead of gelatin, they developed a third polymer component (Figure 15d) [133]. This polymer was modified with norbornene carboxylic acid, which also has photo-crosslinking abilities, allowing it to be stabilized with UV treatment.

## 5. Conclusions

In conclusion, the integration of dynamic covalent bonds into biopolymer materials presents a promising strategy to overcome the inherent limitations of traditional biopolymers, such as poor mechanical properties and uncontrolled degradation rates. This review highlights the significant advancements made in enhancing the functionality of biopolymer networks by incorporating dynamic bonds, including imine, acylhydrazone, and boronate ester, which not only improve mechanical strength and adhesion but also introduce controllable drug release mechanisms. Moreover, the responsiveness of these materials to external stimuli, such as pH, water content, and light irradiation, opens up new possibilities for applications in self-healing, 3D printing, and injectability.

The diverse case studies discussed emphasize the versatility and potential of dynamic covalent bonds in a range of biological applications, from wound healing to drug delivery and bioprinting. Moving forward, research should focus on exploring combinations of different dynamic bonds to create synergistic effects, as well as enhancing the precision with which these materials respond to specific stimuli. Furthermore, efforts should be made to improve the biocompatibility and biodegradability of these biopolymer networks, ensuring their safety for in vivo use and enhancing their environmental sustainability.

Ultimately, dynamic covalent bond technology holds immense promise in driving innovation within the medical, pharmaceutical, and environmental fields. Continued research and development will be crucial in advancing biopolymer materials to meet the growing demands of modern biomedical applications, paving the way for more effective and sustainable therapies.
